# Just 1-min exposure to a pure tone at 100 Hz with daily exposable sound pressure levels may improve motion sickness

**DOI:** 10.1265/ehpm.24-00247

**Published:** 2025-03-25

**Authors:** Yishuo Gu, Nobutaka Ohgami, Tingchao He, Takumi Kagawa, Fitri Kurniasari, Keming Tong, Xiang Li, Akira Tazaki, Kodai Takeda, Masahiro Mouri, Masashi Kato

**Affiliations:** 1 Department of Occupational and Environmental Health, Nagoya University Graduate School of Medicine, Nagoya, Aichi, Japan; 2 Activities of the Institute of Innovation for Future Society of Nagoya University; 3 DENSO CORPORATION, Kariya, Aichi, Japan

**Keywords:** Sound technology, Pure tone, Motion sickness, Vestibular function, Imbalance, Health effect

## Abstract

**Background:**

Motion sickness is a common transportation issue worldwide. Vestibular dysfunction has been reported to be a key etiology of motion sickness. However, there are limited technologies for alleviating motion sickness.

**Methods:**

The most appropriate frequency (Hz) and level (dBZ) of pure tone for modulation of vestibular function were determined by an *ex vivo* study using murine utricle explants. The preventive effects of the selected pure tone on motion sickness were then confirmed by using a beam balance test in mice. The alleviating effects of pure tone on motion sickness induced by a swing, driving simulator or real car were objectively assessed by using posturography and electrocardiography (ECG) and were subjectively assessed by using the Motion Sickness Assessment Questionnaire (MSAQ) in humans.

**Results:**

The effect of short-term (≤5 min) exposure to a pure tone of 80–85 dBZ (= 60.9–65.9 dBA) at 100 Hz on motion sickness was investigated in mice and humans. A mouse study showed a long-lasting (≥120 min) alleviative effect on shaking-mediated exacerbated beam test scores by 5-min exposure to a pure tone of 85 dBZ at 100 Hz, which was *ex vivo* determined as a sound activating vestibular function, before shaking. Human studies further showed that 1-min exposure to a pure tone of 80–85 dBZ (= 60.9–65.9 dBA) at 100 Hz before shaking improved the increased envelope areas in posturography caused by the shakings of a swing, a driving simulator and a vehicle. Driving simulator-mediated activation of sympathetic nerves assessed by the heart rate variable (HRV) and vehicle-mediated increased scores of the MSAQ were improved by pure tone exposure before the shaking.

**Conclusion:**

Since the exacerbated results of posturography and HRV reflect shaking-mediated imbalance and autonomic dysfunction, respectively, the results suggest that the imbalance and autonomic dysregulation in motion sickness could be improved by just 1-min exposure to a pure tone with daily exposable sound pressure levels.

**Trial registration:**

Registration number: UMIN000022413 (2016/05/23–2023/04/19) and UMIN000053735 (2024/02/29–present)

**Supplementary information:**

The online version contains supplementary material available at https://doi.org/10.1265/ehpm.24-00247.

## Graphical Abstract

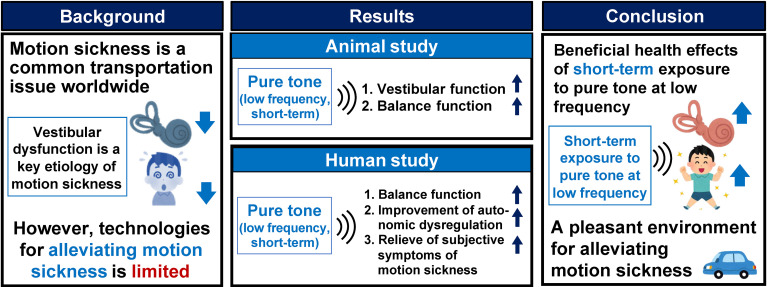

## Introduction

Motion sickness is a common disease in men and women of all ages and has a great impact on the frequency of use of various types of transportation including vehicles, ships, trains and airplanes [1]. The risk of motion sickness in passengers in vehicles is higher than that in drivers [2]. There is therefore a concern about the recent progress in the development of self-driving vehicle technology because drivers will become passengers. However, there are very limited in-vehicle devices that can safely provide a comfortable transportation environment without motion sickness.

An association between vestibular function including otolith reflex pathways and motion sickness has been suggested in previous studies [3–8]. Beam tests have been used for semi-quantitative evaluation of balance function in animal studies on motion sickness [4–6]. Assessment of the envelope area by posturography has been used for quantitative evaluation of imbalance caused by motion sickness in human studies [9, 10]. Autonomic dysregulation following imbalance has also been reported as a typical symptom of motion sickness [11–13]. Previous studies on heart rate variability (HRV) revealed that a component of the normalized high-frequency power (HF) and the ratio of low-frequency to high-frequency power (LF/HF ratio) could be markers for parasympathetic nerve activity and sympathetic nerve activity, respectively [14, 15]. Therefore, autonomic dysregulation can be objectively assessed by the HF and LF/HF ratio by HRV in humans. Furthermore, the motion sickness assessment questionnaire (MSAQ) is a useful tool to comprehensively evaluate the subjective symptoms derived from motion sickness in humans [16, 17].

Previous studies physiologically indicated that vibration at 100 Hz could activate not only otolithic functions but also canal afferents [18]. Previous studies morphologically showed that exposure to a pure tone at 100 Hz could modulate vestibular function by affecting the otolith in the inner ears of mice [19–21]. A previous epidemiological study for healthy people showed that exposure to a sound component of 100 Hz in music improved balance function assessed by the envelope area in posturography [22]. These results in mice and humans suggest that the pure tone at 100 Hz can modulate vestibular function. To our knowledge, however, the ranges of frequencies and sound pressure levels of functional sounds that can activate vestibular function have not been clarified.

Vestibular dysfunction-mediated imbalance and subsequent autonomic dysregulation are key etiologies for motion sickness [3–6]. Therefore, a sound that can activate vestibular function may alleviate motion sickness. To validate the hypothesis, we carried out an *ex vivo* study in mice for screening frequencies (90–1000 Hz) and sound pressure levels (65–85 dBZ) of pure tones that activate vestibular function of the mouse inner ear. We then carried out *in vivo* studies in mice and humans to clarify whether the selected pure tone with daily exposable sound pressure levels could mitigate motion sickness with focus on imbalance and/or subsequent autonomic dysregulation caused by different kinds of shaking. This study focused on motion sickness induced by side-to-side and up-and-down motions associated with otolithic function rather than by rotation involved in semicircular canal, with the aim of mimicking the shaking of a vehicle. This is the first attempt to create a more comfortable transportation environment with mitigation of motion sickness.

## Methods

### Ethical permission for studies in mice and humans

Our animal experiments were approved by the Institutional Animal Care and Use Committee in Nagoya University (approval number: M220179-001). This study was conducted and reported in accordance with the Japanese Government Regulations for Animal Experiments and the ARRIVE guidelines.

All studies involving human participants have been conducted according to the principles of the Declaration of Helsinki. The purpose and procedures of this research were explained in person to each participant. Participants were informed that they could withdraw from participation at any time without any justification. Written informed consent was obtained from all participants prior to inclusion in the study. The human study was approved by the Ethics Committee of Nagoya University School of Medicine (2016-0036) and registered in the University Hospital Medical Information Clinical Trials Registry (UMIN-CTR) system [Registration number: UMIN000022413 (May 23, 2016–April 19, 2023) and UMIN000053735 (Feb. 29, 2024–present)].

### Mouse studies

An *ex vivo* study using FM1-43FX fluorescent dye (Invitrogen, Thermo Fisher Scientific, USA) in an explant culture of murine utricles from 5–9-day-old ICR mice (Japan SLC, Inc.) in the presence or absence of exposure to various pure tones was conducted following a method previously described [23]. Ion channel activity in the utricles, which corresponds to vestibular function [23, 24], was determined by the fluorescent levels of FM1-43FX uptake for 10 sec just after the *ex vivo* sound exposure.

**Fig. 1:**
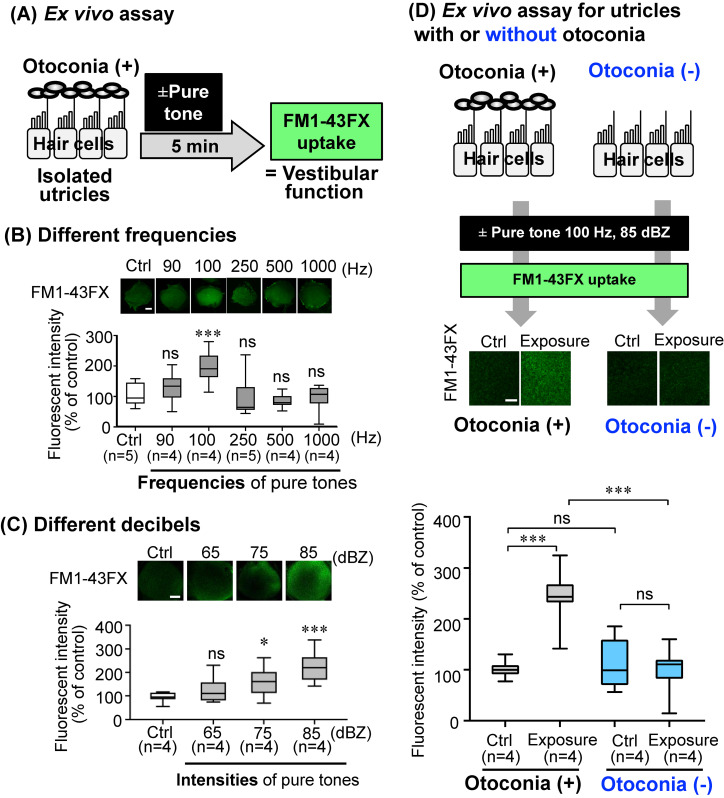
Screening of pure tones that activate ion channel function in explant culture of the utricles. (A) The protocol of an *ex vivo* experiment of explant culture for the murine utricle in the presence or absence of exposure to a pure tone for 5 min is presented. The *ex vivo a*ssay for ion channel activities in utricles, an index of vestibular function, determined by the fluorescent levels of FM1-43FX uptake for 10 sec following the method previously shown [23, 24]. The utricles were treated with FM1-43FX fluorescent dye just after *ex vivo* sound exposure. (B, C) The FM1-43FX fluorescent intensities in the utricles after exposure to a pure tone of 85 dB at different frequencies (90–1,000 Hz) for 5 min (B) and exposure to a pure tone at 100 Hz with different sound pressures (65–85 dBZ) for 5 min (C) were compared to those without pure tone exposure (Ctrl). (D) Effects of a pure tone on ion channel function in explant culture of utricles with otoconia [otoconia (+)] or without otoconia [otoconia (−)] are presented. Utricles without otoconia were prepared just after removal of otoconia by using a fine toothbrush following a method previously reported [23]. The fluorescent intensities after exposure to the pure tone of 85 dB at 100 Hz for 5 min followed by FM1-43FX uptake for 10 sec were compared to those without pure tone exposure (Ctrl) in utricles with or without otoconia. Scale bars: 100 µm. Significant differences (*P < 0.05, ***P < 0.001) and not significant differences (ns) compared with the control were analyzed by pairwise comparison after the Kruskal-Wallis *H* test.

**Fig. 2:**
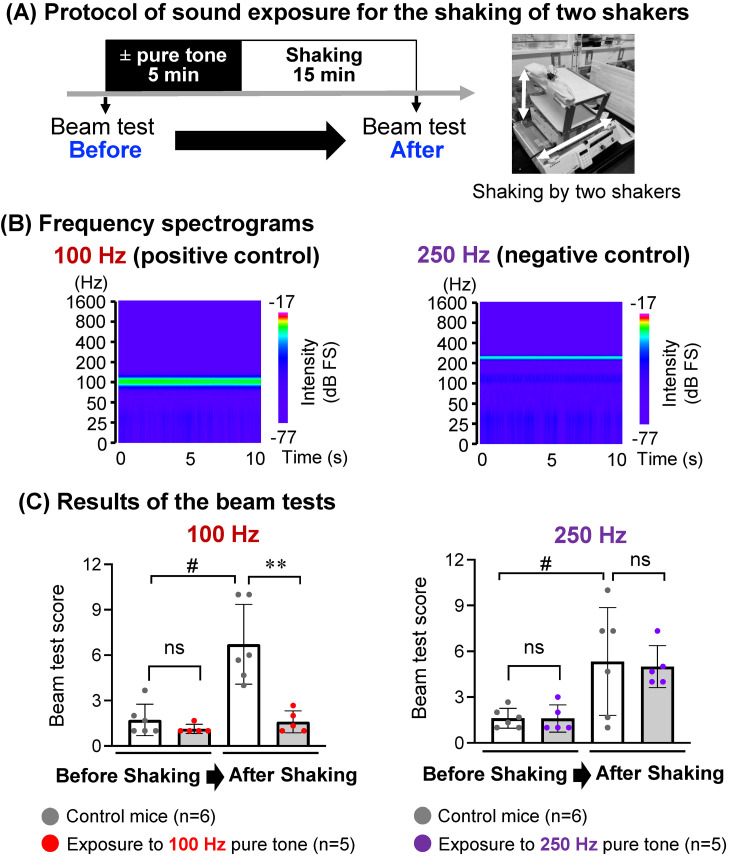
Effects of short-term exposure to pure tones on motion sickness in mice. (A) The experimental protocol for the *in vivo* study in the presence or absence of sound exposure for 5 min just before vertical (50 rpm) and horizontal (80 rpm) shaking by 2 shakers for 15 min (Suppl. Fig. S2A) is presented. (B) Frequency spectrograms of pure tones at 100 Hz (left) and 250 Hz (right) to which wild-type mice were exposed according to a previously reported method are shown [26]. (C) Scores of beam tests are presented by graphs of boxplots. Beam tests before shaking and just after shaking were carried out for mice (n = 5–6 in each group) with exposure to a pure tone of 85 dBZ at 100 Hz or 250 Hz or without sound exposure (Control mice). Significant differences (**P < 0.01) and not significant differences (ns) were analyzed by the Mann-Whitney *U* test; ^#^*P* < 0.05 was analyzed by Wilcoxon’s signed-rank test.

**Fig. 3:**
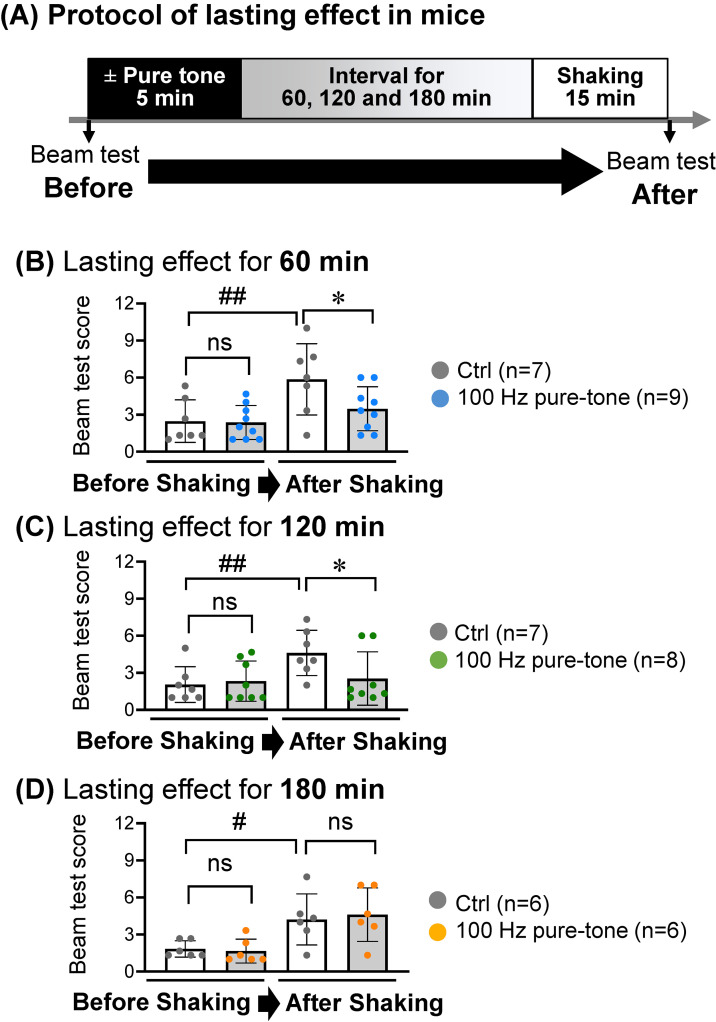
Lasting effect of a pure tone at 100 Hz on motion sickness in mice. (A) The experimental protocol for an *in vivo* study in the presence or absence of 5-min exposure to a pure tone of 85 dBZ at 100 Hz before vertical and horizontal shaking of shakers for 15 min (Suppl. Fig. S1A) is presented. (B–D) Beam tests before sound exposure (Before) and at 60 min (B), 120 min (C) and 180 min (D) after shaking were carried out for mice with exposure to a pure tone of 85 dBZ at 100 Hz (100 Hz) or without sound exposure (Ctrl). Scores of beam tests are presented by graphs of boxplots. Significant differences (*P < 0.05) and not significant differences (ns) were analyzed by the Mann-Whitney *U* test; ^#^*P* < 0.05 and ^##^*P* < 0.01 were analyzed by Wilcoxon’s signed-rank test.

Beam balance tests using ICR mice at 3–4 months of age were conducted as in our previous study [25]. Exposure to pure tones was performed following the methods used in a previous study with slight modifications [26]. Briefly, each mouse was exposed to the pure tone of 85 dBZ at 100 Hz or 250 Hz in a cage. The pure tone was exposed from the top side by using a KSC-SW11 speaker (KENWOOD, Japan) that was approximately the same size as the cage. The distance between the mouse and the speaker was 15 cm. The sound pressure level was measured at the head of a mouse, which was fixed in a sack during shaking in a cage, but not at the speaker. Two shakers shaking in different directions (vertical: 50 rpm, horizontal: 80 rpm) were used to induce motion sickness in mice. More information on the experimental protocols for mice is diagrammatically shown in Figs. 1–3 and Supplementary Methods.

### Participants in human studies

The participants in human studies were recruited through posters in Nagoya University campus. The health condition and vestibular function of the subjects were confirmed by self-reporting. Participants with a history of motion sickness were included, while those with hearing loss or vertigo/dizziness were excluded. Each subject signed an informed consent form prior to participation and the subjects were asked not to drink coffee or tea and not to take motion sickness-related drugs including antihistamine during the experimental period. The number of participants in each trial (swing, driving simulator and vehicle) was set on the basis of previous studies in which motion sickness in humans was evaluated [27–30]. As shown in Suppl. Tables S1-1 and S1-2, a total of 82 participants registered in the UMIN system participated in plural trials. Basic information for participants in different trials is shown in Suppl. Table S2. A self-controlled design was used for all human study trials. Participants were assigned to a group without pure tone exposure and a group with pure tone exposure in each trial. Recovery time between trials with or without pure tone exposure was determined to ensure baseline consistency. Detailed information for each trial is outlined in the figures.

### Shaking of a swing

**Fig. 4:**
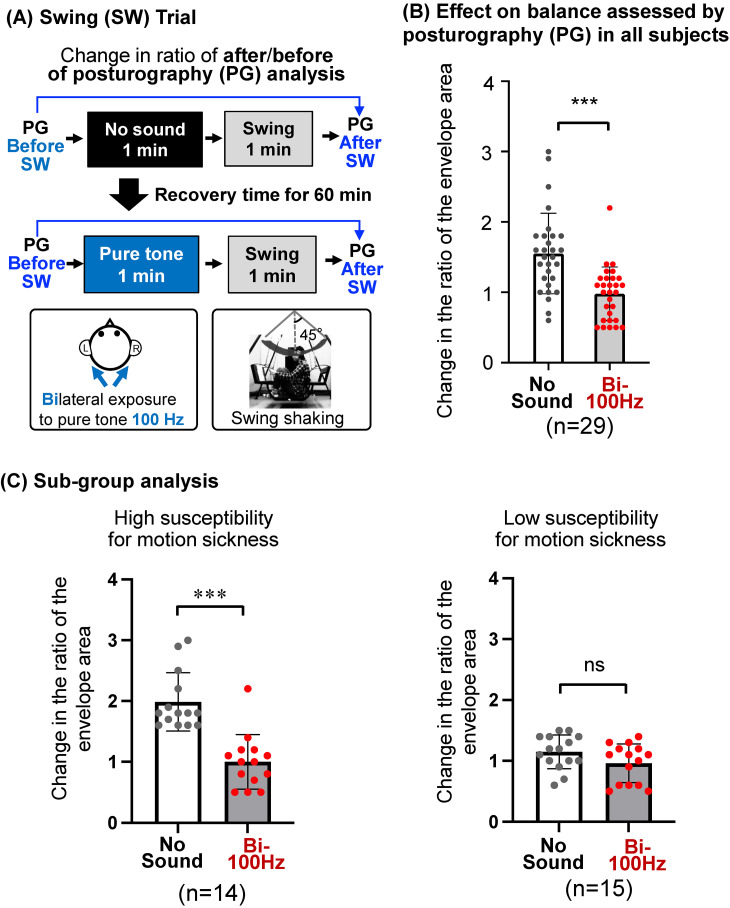
Effect of a pure tone on motion sickness caused by a swing in humans. (A) The experimental protocol for balance assessed by posturography in the absence or presence of 1-min exposure to pure tone of 85 dBZ at 100 Hz just before 1-min swing shaking is presented with the setting of the swing and the method used for pure tone exposure. (B) Change ratios of the envelope area evaluated by posturography before and after the shaking in all subjects with bilateral (Bi-100 Hz, n = 29) exposure to the pure tone at 100 Hz before the shaking were compared to those in the same subjects without sound exposure (No sound). (C) Sub-group analysis of effect of pure tone on balance in subjects with different sensitivities for motion sickness. Subjects (n = 14) with high susceptibility (n = 14) or with low susceptibility (n = 15) to motion sickness were divided by the median of the change ratio of envelope area of posturography before and after the shaking. Changes in the ratio of the envelope area evaluated by posturography before and after the shaking in subjects with bilateral exposure to the pure tone before the shaking were compared to those in the same subjects without sound exposure (No sound) in the high (left graph) and low (right graph) susceptibility groups. Significant differences (***P < 0.001) and not significant differences (ns) were analyzed by the paired *t*-test. SW, swing; PG, posturography.

Human subjects were shaken by a swing (Blanco-Europe, Tsuyoni, Japan) for 1 min as shown in Fig. 4A. Briefly, the swing was manually shaken at a speed of 30 rpm (0.5 Hz) with a shaking angle of 45 degrees. The subjects were given the task of reading a document about a tale during the shaking, since it has been reported that the severity of motion sickness in adults is increased by reading a book in a moving vehicle [31].

### Shaking of a driving simulator

An immersive driving simulator system in Nagoya University, which has a 360° 3D road and traffic environment screen (UC-win/Road, FORUM8 Corporation, Japan) integrated with a Cruise in AVL InMotion system (Powered by CarMarker), was used for the shaking of a driving simulator. Six degrees-of-freedom of motion control enabled us to produce the motion of the driving simulator to match that of the 3D road screen. The setting was automated driving at 40 km/h for 3 min.

### Shaking of a real vehicle

Each participant was asked to sit in the rear seat of a vehicle and to silently read a document about a tale during the vehicle trial to induce motion sickness. Participants were instructed to briefly recount the tales after the trial to confirm their active engagement in reading during the experiment in the vehicle. The vehicle was moved for 3 min under the following stop and go conditions in a closed test course with two straight segments joined by curves at both ends: moving forward for 15 m at 10 km/h and then stopping for 1 sec, moving forward for 20 m at 20 km/h and then stopping for 1 sec, moving forward for 15 m at 15 km/h and then stopping for 1 sec, moving forward for 25 m at 25 km/h and then stopping for 1 sec, moving forward for 20 m at 20 km/h and then stopping for 1 sec, moving forward for 30 m at 25 km/h and then stopping for 1 sec, moving forward for 15 m at 15 km/h and then stopping for 1 sec and moving forward for 5 m at 10 km/h.

### Exposure to pure tones in humans

Exposure of humans to pure tones was performed as in our previous study with slight modifications [26]. Briefly, our sound exposure system consisted of a generator (WF1947, NF, Japan), an amplifier (AI-301DA-X/B, TEAC, Japan) and a KSC-SW11 speaker or an SL-D501 speaker (ONKYO, Japan). The SL-D501 speaker were used during the shaking of a swing and the shaking of a driving simulator, and the KSC-SW11 speaker was used during the shaking of a vehicle. Bilateral sound exposure from the occipital sides was performed with the SL-D501 speaker at equal distances (30 cm) from the right and left inner ears in the swing and driving simulator trials. Unilateral sound exposure from the right side was performed with the speaker at a distance of 30 cm from the right inner ear in the swing trial. Two KSC-SW11 speakers toward the right and left inner ears were used in the vehicle trial. Each speaker at the headrest position of a vehicle seat was 10 cm from the right or left inner ear. Participants had no knowledge about the significance of unilateral or bilateral exposure to pure tones in this study. Although a few different kinds of sound exposure systems were used in this study, the intensities (dBZ) and frequencies (Hz) of pure tones at the temporal areas, where the inner ears are housed, were accurately measured in participants using a precision sound meter (TYPE 6236 with an FFT analyzer, ACO CO., LTD, Japan). In order to ensure the safety of participants, 1-minute exposure to pure tones of ≤85 dBZ, which is lower than the levels typically encountered in urban and workplace areas, was used [32–35]. In fact, no participant reported any complains about the sound level of the pure tone. Participants could quit the trial at any time if they felt discomfort. Staff members with a medical background were assigned to observe the health condition of participants during the trial to ensure their safety.

### Evaluation of imbalance caused by shaking in humans

Imbalance in participants without exposure to the pure tone and that in participants with exposure to the pure tone before and just after the shaking were assessed by the envelope areas in posturography. The method for posturography in this study was the same as the method used in previous studies [10, 22, 36]. Static posturography was conducted by using a force plate (Version 2.03, Universal Medical Communication, Japan) at a sampling frequency of 20 Hz. The envelope area of movement of the subject’s center of gravity under the condition of eyes closed, arms at each side of the body, and legs and feet together was measured for 1 min [22]. To decrease the effect of individual differences, the change in the ratio based on the self-comparison of each participant was used for the assessment of imbalance.

### Evaluation of autonomic dysregulation caused by shaking in humans

Autonomic dysregulation following vestibular dysfunction [11–13] in participants with 1-min exposure to the pure tone and that in participants without exposure to the pure tone before and after the shaking of a driving simulator for 3 min were evaluated by HRV. HRV was measured by a portable device for electrocardiography (ECG) (CheckMyHeart 3.0, Daily Care, BioMedical Inc.) by the method used in previous studies [37, 38]. The change in the ratio based on the self-comparison of each participant was used for the assessment of autonomic dysregulation for reducing the impact of individual differences.

### Evaluation of subjective symptoms caused by shaking in humans

Subjective symptoms in participants without exposure to the pure tone and same participants with exposure to the pure tone before and just after the shaking of a vehicle were assessed by the MSAQ as shown previously [16, 17]. The MSAQ can be used for evaluation of overall subjective symptoms of motion sickness by total scores as well as evaluation of specific subjective symptoms categorized into gastrointestinal, central, peripheral and sopite-related subscale scores [16]. A higher MSAQ score indicates more severe motion sickness symptoms. The self-comparison of each participant was used for the assessment of subjective symptoms to reduce the effect of individual differences. The history of motion sickness was investigated using a simple yes/no question.

### Statistical analysis

All analyses were carried out by using SPSS version 24.0 (IBM. Inc. USA.) and GraphPad Prism 9. Normally distributed data were analyzed by the paired *t*-test [39], while non-normally distributed data were analyzed by the Mann-Whitney *U* test [40], Kruskal-Wallis *H* test [41] and Wilcoxon’s signed-rank test [42], which are shown in the section of figure legends, following previous studies. All statistical tests were two-sided and *P* < 0.05 was considered as statistically significant.

## Results

### *Ex vivo* screening of frequencies and sound pressure levels that activate vestibular function in mice

An *ex vivo* mouse study for evaluating ion channel function in an explant culture of a murine utricle has been reported to be useful to identify stimulations that can activate vestibular function [23, 24, 43]. An *ex vivo* mouse study using sundry sound frequencies from 90 Hz to 1000 Hz and various sound pressure levels from 65 dBZ to 85 dBZ showed that short-term (5 min) exposure to a pure tone at 100 Hz activates vestibular function (Fig. 1A–C). Activated vestibular function in the utricle with intact otoconia via exposure to a pure tone of 85 dBZ at 100 Hz was attenuated in the utricles without otoconia (Fig. 1D).

### Effect of exposure to pure tones on imbalance caused by vertical and horizontal shaking in mice

According to our *ex vivo* mouse study, a pure tone of 85 dBZ at 100 Hz and a pure tone of 85 dBZ at 250 Hz were selected as a positive control and a negative control, respectively. An *in vivo* mouse study (Fig. 2A) was then conducted to determine whether short-term exposure to a pure tone can improve imbalance as a symptom of motion sickness induced by shaking that approximately mimicks the shaking of a vehicle (Suppl. Fig. S1AD). The effects of 5-min exposure to a pure tone of 85 dBZ at 100 Hz (Fig. 2B, left) or 250 Hz (Fig. 2B, right) just before shaking for 15 min on imbalance as a symptom of motion sickness were evaluated by scores of beam tests, an index for balance function in mice [4–6]. The scores of beam tests just after the shaking were significantly (*P* < 0.05) higher than those before shaking in mice without sound exposure, indicating the development of motion sickness. The increased scores due to shaking were improved by 5-min exposure to a pure tone at 100 Hz (Fig. 2C, left) but not by 5-min exposure to a pure tone at 250 Hz (Fig. 2C, right) just before shaking for 15 min. Furthermore, 5-min exposure to a pure tone of 85 dBZ at 100 Hz with intervals of 60 min and 120 min (Fig. 3ABC) but not with an interval of 180 min (Fig. 3AD) before the same shaking (Suppl. Fig. S1AD) for 15 min significantly decreased the scores of beam tests.

### Effect of a pure tone at 100 Hz on imbalance caused by a swing in humans

A swing was used for shaking that approximately mimicked the shaking of a vehicle (Suppl. Fig. S1BD). Individual results of the change in the ratio of the envelope area after the shaking were significantly increased compared to those before the swing shaking in the same subjects without exposure to the pure tone (Suppl. Fig. S2). These results for human subjects without pure tone exposure indicate the development of imbalance, a symptom of motion sickness, by swing shaking for 1 min.

After establishing the swing-mediated imbalance, the effects of exposure to a pure tone of 85 dBZ at 100 Hz for 1 min just before shaking for 1 min on balance were investigated by the change in the ratio of envelope areas in posturography in humans (Fig. 4A). The change in the ratio of the envelope area after shaking (Fig. 4B) in subjects with bilateral exposure of equal stimulation toward both inner ears to the pure tone at 100 Hz (Bi-100 Hz) was significantly smaller than that in the same subjects without sound exposure (No sound). These results indicate improvement of swing-mediated imbalance by the pure tone exposure. Subgroup analysis of high- and low-susceptibility subjects for motion sickness showed that bilateral exposure to the pure tone at 100 Hz significantly decreased the changes in the ratio of envelope areas in the high-susceptibility subjects (Fig. 4C, left) but not in the low-susceptibility subjects (Fig. 4C, right). Not only unilateral exposure of one side stimulation toward the right inner ear to the pure tone at 100 Hz (Suppl. Fig. S3ABC) but also bilateral exposure to the pure tone at 250 Hz (Suppl. Fig. S3ABD) had limited effects on the ratios of the envelope area before and after swing shaking. These results suggest that bilateral exposure to a pure tone of 85 dBZ at 100 Hz is useful for alleviating the imbalance caused by shaking of a swing in humans.

### Effects of a pure tone at 100 Hz on imbalance and autonomic dysregulation by shaking of a driving simulator in humans

The shaking caused by a driving simulator was similar to the shaking caused by a vehicle (Suppl. Fig. S1CD). Individual results for the change in the ratios of the envelope area (Suppl. Fig. S4A) and LH/HF ratio (Suppl. Fig. S4B, right), an indicator of sympathetic nerve activity, after the shaking were significantly higher than those before the shaking in the same subjects without exposure to the pure tone. In contrast, individual results for change in the ratio of HF (Suppl. Fig. S4B, left), an indicator of parasympathetic nerve activity, were significantly lower than those before the shaking in the same subjects without exposure to pure tone. These results for human subjects without pure tone exposure suggest that imbalance and activation of sympathetic nerves as symptoms of motion sickness were caused by the shaking a driving simulator for 3 min.

**Fig. 5:**
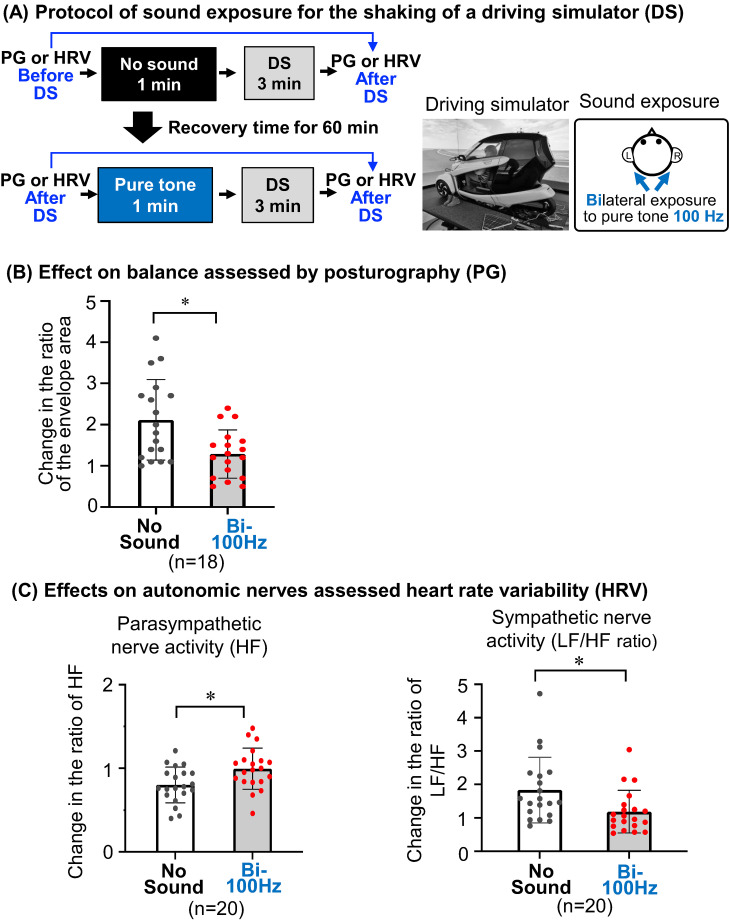
Effects of a pure tone on motion sickness caused by a driving simulator in humans. (A–C) The experimental protocol (A) to assess the balance by posturography (B) or autonomic dysregulation by heart rate variability (HRV) (C) in the absence or presence of 1-min exposure to pure tone of 85 dBZ at 100 Hz just before 3-min driving simulator shaking is presented with the setting of the driving simulator and the method used for pure tone exposure. (B, C) Change ratios of the envelope area evaluated by posturography (B) and parasympathetic nerve activity (HF) and sympathetic nerve activity (LF/HF ratio) assessed by HRV (C) before and after the shaking in subjects with bilateral (Bi-100 Hz) exposure to the pure tone before the shaking were compared to those in the same subjects without sound exposure (No sound). The results are presented in graphs of boxplots with individual values. Significant differences (*P < 0.05) were analyzed by the paired *t*-test. DS, driving simulator; PG, posturography; HR, heart rate variability.

After establishing the driving simulator-mediated imbalance and autonomic dysregulation, the effects of bilateral exposure to a pure tone of 85 dBZ at 100 Hz for 1 min before shaking by an automated driving simulator for 3 min (Fig. 5A) on imbalance (Fig. 5B) and autonomic nerves (Fig. 5C) were then evaluated by changes in the ratios of envelope areas in posturography and changes in the ratio of HF and LF/HF ratio assessed by HRV, respectively. The change in the ratio of the envelope area after shaking in subjects with bilateral exposure to the pure tone before the shaking was significantly lower than that in the same subjects without sound exposure (Fig. 5B). Moreover, the change in the ratio of HF after shaking in subjects with bilateral exposure to the pure tone at 100 Hz was significantly higher than that in the same subjects without sound exposure (Fig. 5C, left). The change in the ratio of LF/HF ratio after shaking in subjects with bilateral exposure to the pure tone at 100 Hz was significantly lower than that without sound exposure in the same subjects (Fig. 5C, right). These results suggest that imbalance and activation of sympathetic nerves triggered by the shaking of a driving simulator were alleviated by exposure to the pure tone before the shaking.

### Effects of a pure tone at 100 Hz on objective and subjective symptoms of motion sickness caused by a vehicle in humans

Following the results obtained by the shaking of a driving simulator, it was finally investigated whether the pure tone could alleviate motion sickness caused by a real vehicle. Individual results for the change in the ratios of the envelope area after the shaking of a vehicle for 3 min were significantly higher than those before the shaking in the same subjects without pure tone exposure (Suppl. Fig. S5). These results for human subjects without pure tone exposure indicate the development of imbalance by vehicle shaking for 3 min.

**Fig. 6:**
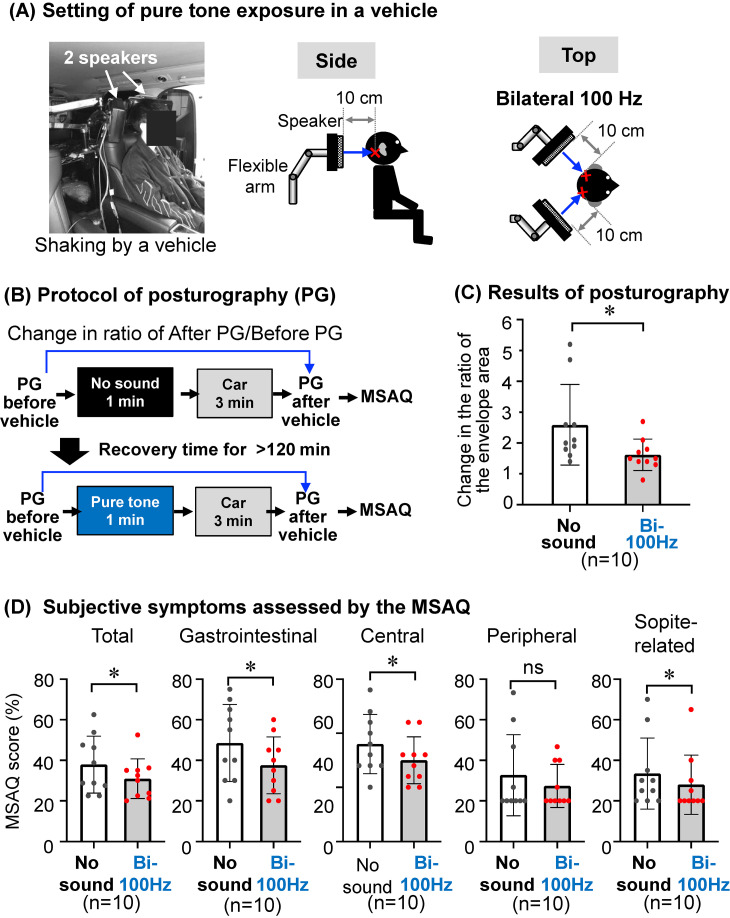
Effects of a pure tone on motion sickness caused by a vehicle in humans. (A) A photograph (left) and two schemas viewed from the side (center) and top (right) for the setting of exposure to a pure tone from occipital sides by two speakers with flexible arms in a vehicle are presented. The sound pressure level (80 dBZ) of the pure tone at 100 Hz was measured 10 cm from the speakers at the temporal areas (red cross marks), where the inner ears are housed. (B–D) The experimental protocol (B) to assess the balance by posturography (C) and to the assess subjective symptoms by the motion sickness assessment questionnaire (MSAQ) (D) in subjects with bilateral (Bi-100 Hz) exposure to the pure tone before the shaking were compared to those in the same subjects without sound exposure (No sound). Significant differences (*P < 0.05, **P < 0.01) and not significant differences (ns) were analyzed by the paired *t*-test. PG, posturography; MSAQ, motion sickness assessment questionnaire.

A sound system designed to deliver a pure tone of 80 dBZ at 100 Hz toward the temporal regions, where the inner ears are located, was installed at the headrest of a vehicle seat (Fig. 6A). The effects of bilateral exposure to the pure tone of 80 dBZ at 100 Hz for 1 min from the occipital side before 3-min shaking by travel motion of a vehicle on imbalance in motion sickness (Fig. 6AB) were evaluated by the ratios of envelope areas in posturography (Fig. 6C). The change in the ratio of the envelope area after shaking in subjects with bilateral exposure to the pure tone at 100 Hz (Fig. 6C, Bi-100 Hz) was significantly lower than that in the same subjects without sound exposure (Fig. 6C, No sound). The effects of the pure tone exposure before shaking by the travel motion on subjective symptoms (Fig. 6BD) were also investigated by the scores of an MSAQ that was used in previous studies [16, 17]. The MSAQ was completed after the posturography test. Since the duration of the posturography measurement is only 1 minute, its impact on the MSAQ results is expected to be minimal. The total MSAQ score and scores for most of the items in the MSAQ after shaking in subjects with pure tone exposure (Fig. 6D, Bi-100 Hz) were significantly lower than those for the same subjects without pure tone exposure (Fig. 6D, No sound).

These results suggest that imbalance and most of subjective symptoms caused by the shaking of a vehicle were mitigated by the pure tone exposure before the shaking.

## Discussion

Our *ex vivo* screening study in mice for sound pressure levels (65–85 dBZ) and frequencies (90–1000 Hz) of pure tones revealed that pure tones of 75–85 dBZ at 100 Hz are specific sounds that activate vestibular function. Since the pure tone activated vestibular function in utricles with intact otoconia but not in utricles without otoconia, otoconia may be a potential target of the pure tone. Since vestibular function has been shown to play a crucial role in motion sickness [3, 44], the mitigating effect of pure tone on motion sickness was investigated in this study. Our mouse study showed that 5-min exposure to a pure tone of 85 dBZ at 100 Hz (positive control) but not at 250 Hz (negative control) with intervals of 60 min and 120 min but not with an interval of 180 min before shaking significantly improved the beam test scores, which are an index of balance function [4–6]. These results suggest long-lasting (≥120 min) preventive effects of short-term exposure to a pure tone of 85 dBZ at 100 Hz before shaking on shaking-mediated imbalance by activation of vestibular function via otoconia.

Considering the convenience and safety of daily life as well as the results of our mouse studies, we further investigated the effects of a 1-minute exposure to a pure tone at 100 Hz on imbalance caused by the shaking of a swing, a driving simulator or a vehicle in humans. Exposure to equal stimulation of the right and left inner ears with pure tones at 80–85 dBZ and 100 Hz prior to shaking induced by shaking with a swing, driving simulator or vehicle significantly reduced the increase in envelope areas in posturography. However, the effects of unilateral exposure to a pure tone of 85 dBZ at 100 Hz toward the inner ear on one side (right) (Suppl. Fig. S3C) and bilateral exposure to a pure tone of 85 dBZ at 250 Hz on the swing-induced increase in envelope areas in posturography were limited. These results suggest that the exposed sound should meet all of the following conditions to alleviate the imbalance in motion sickness: 1) equal exposure to the inner ears on both sides, 2) a pure tone with a frequency of 100 Hz, 3) a sound level of 80–85 dBZ measured at the temporal regions in which the inner ears are housed and 4) a stable pure tone duration of 1 minute. Since it is rare to encounter environmental sounds that strictly meet these conditions in our daily life, the special sound stimulation device proposed in this study may be necessary to prevent motion sickness. Together with the results of our *ex vivo* mouse study on the utricle, these findings suggest that the pure tone at 100 Hz may mitigate both subjective and objective symptoms of motion sickness, potentially by targeting otoconia. Further studies are necessary to obtain more direct evidence.

Autonomic dysregulation triggered by imbalance in motion sickness, resulting in induction of sundry symptoms [11–13], was investigated in this study. The results for HRV showed that decreased HF and increased LF/HF ratios caused by shaking by a driving simulator were increased and decreased, respectively, by 1-min exposure to the pure tone before the shaking of a driving simulator. These results suggest that activation of sympathetic nerves, an autonomic dysregulation in motion sickness, was objectively improved by the pure tone exposure. In addition, most of subjective symptoms evaluated by MSAQ scores taking imbalance and autonomic dysregulation in motion sickness into account [16] were decreased by bilateral exposure to the pure tone before travel motion. Our findings suggest that pure tone has a mitigating effect on both autonomic dysfunction and inner ear dysfunction. If a pure tone can directly improve inner ear function and/or autonomic function, it may mitigate other diseases associated with these dysfunctions. Indeed, our previous study demonstrated that Meniere’s disease was alleviated by exposure to a pure tone at 100 Hz [45]. Further research is required to identify the specific targets and mechanisms of pure tone action for broader preventive applications.

In World Health Organization (WHO) regulations, an equivalent sound level of 85 dBA (= 104 dBZ of pure tone at 100 Hz) for 480 min is a regulatory limitation of occupational exposure [46]. An exposed sound pressure level of 80–85 dBZ (= 60.9–65.9 dBA) of pure tone at 100 Hz is about 20 dBZ lower than the limitation of the equivalent sound level. More importantly, the effect of exposure to a pure tone for only 1 min on the equivalent sound level for 480 min is very limited. Furthermore, exposure to environmental sounds containing pure tone at 100 Hz with a sound pressure level of 80–85 dBZ is common in daily human life, because the sound pressure level approximately corresponds to the level in our usual conversation [47, 48]. The results of analysis of distortion product otoacoustic emissions (DPOAEs) also indicated no effect on hearing levels at 4k Hz by 1-min exposure to a pure tone of 85 dBZ at 100 Hz in humans (Suppl. Fig. S6). The multidirectional information indicates that the health risk of short-term exposure to a pure tone of 80–85 dBZ at 100 Hz is limited.

## Conclusion

The results of our human study with a self-control design suggested that short-term exposure to a pure tone of 75–85 dBZ at 100 Hz, which was determined to be an activator of vestibular function via otoconia in mice, before shaking alleviates the objective and subjective symptoms for imbalance and subsequent autonomic dysregulation in motion sickness. A long-lasting alleviative effect on motion sickness in humans is expected by short-term exposure to the pure tone because its mitigative effect on imbalance caused by shaking could continue for ≥120 min in mice. Motion sickness derived from a vehicle as well as that derived by a swing and a driving simulator in humans were improved by the pure tones at 100 Hz that maintained a sound pressure level of 80–85 dBZ at the temporal areas, even though the sound exposure systems were slightly different. Taken together, the results indicate that the pure tone technology may have reached a level that is near the level for practical use to develop a pleasant environment for preventing motion sickness in various types of transportation. In the next step, the effects of pure tone exposure during a motion event on motion sickness need to be investigated.

## Abbreviations

DPOAE: Distortion Product Otoacoustic Emission
ECG: Electrocardiography
HF: High-frequency power
HRV: Heart rate variable
LF: Low-frequency power
MSAQ: Motion Sickness Assessment Questionnaire

## Supplementary information

**Additional file 1:** **Supplementary Methods**.

**Supplemental Figure S1.** Movements of mice and human heads monitored by 6-axis motion sensors. **Supplemental Figure S2.** Imbalance caused by a swing (SW) in human subjects without pure tone exposure. **Supplemental Figure S3.** Effects of different pure tone exposures on motion sickness caused by a swing in humans. **Supplemental Figure S4.** Imbalance and autonomic dysregulation caused by a driving simulator in humans without pure tone exposure. **Supplemental Figure S5.** Imbalance caused by a vehicle in human subjects without pure tone exposure. **Supplemental Figure S6.** Effects of exposure to a pure tone on hearing levels in humans. **Supplemental Table S1.** List of subjects participated in different experiments. **Supplemental Table S2.** Basic characteristics of participants.

## Declarations

### Availability of data and materials

Data available on request from authors.
