# Supplementary material for: Just 1-min exposure to a pure tone at 100 Hz with daily exposable sound pressure levels may improve motion sickness
Source: Environ Health Prev Med. 2025 Mar 25;30:22. doi: 10.1265/ehpm.24-00247 (PMC11955832; doi:10.1265/ehpm.24-00247)
Supplement: Supplementary file 1 — Additional file 1: Supplementary Methods. [file ehpm-30-022-s001a.docx]

**Supplementary information**

**Just 1-min exposure to a pure tone at 100 Hz with daily exposable**

**sound pressure levels may improve motion sickness**

Yishuo Gu^1^, Nobutaka Ohgami^1^, Tingchao He^1^, Takumi Kagawa^1^, Fitri Kurniasari^1^, Keming Tong^1^, Xiang Li ^1^, Akira Tazaki^1,2^, Kodai Takeda^3^, Masahiro Mouri^2,3^ and

Masashi Kato^1,2,*^

^1^Department of Occupational and Environmental Health, Nagoya University Graduate School of Medicine, Nagoya, Aichi, Japan. ^2^Activities of the Institute of Innovation for Future Society of Nagoya University. ^3^DENSO CORPORATION, Kariya, Aichi, Japan.

*Corresponding author: Masashi Kato, MD, PhD,

Department of Occupational and Environmental Health,

Nagoya University Graduate School of Medicine, 65 Tsurumai-cho, Showa-ku, Nagoya, Aichi 466-8550, Japan. Phone: +81-52-744-2122. Fax: +81-52-744-2124.

e-mail: kato.masashi.r6@f.mail.nagoya-u.ac.jp

**Supplementary Methods**

**Animal study**

Male ICR mice (Japan SLC, Inc.) at 3-4 months of age were kept under a specific pathogen-free (SPF) condition with temperature of 23 ± 2˚C, humidity of 55 ± 10% and a 12-h light and dark cycle. The mice had free access to standard mouse chow and water.

***Ex vivo* experiments**

Utricles were dissected from 5-9-day-old ICR mice, and they were put into HBSS buffer (Wako, Japan) in a 6-well dish. Then the utricles were treated with 5 μM FM1-43FX for 10 sec at room temperature. The fluorescence intensities of utricles in the presence and absence of otoconia that were removed by toothbrush hair under a microscope were observed by using an LSM880-ELYRA microscope (Carl Zeiss Meditec AG, Germany) and analyzed by ImageJ.

**Movements monitored by a 6-axis motion sensor**

Movement of the whole body of each mouse during the shaking by two shakers (Suppl. Fig. S1A) and movement of the head of each subject during the shaking by a swing (Suppl. Fig. S1B), a driving simulator (Suppl. Fig. S1C) and a vehicle (Suppl. Fig. S1D) were monitored by a 6-axis motion sensor (MPU-6050, TDK InvenSense, Japan) at a sampling rate of 20 Hz. Acceleration speeds of the mouse and human head movements in X, Y and Z directions and angular velocities in roll, yaw and pitch were recorded.

**Heart rate variability**

Heart rate variability was measured by using a single-lead ECG recorder (Lead I) with a 4-ms resolution of the R-R interval by the method used in previous studies [1, 2]. Briefly, two skin electrodes were placed at the inner sides of the forearms of each subject. The subjects were instructed not to move or talk during the recording for 5 min. Measurements were performed between 10 a.m. and 16 p.m. in a quiet room with a temperature of about 24℃. Normalized low-frequency power (LF power: 0.04-0.15 Hz), high-frequency power (HF power: 0.15-0.4 Hz) and the ratio of low-frequency to high-frequency power (LF/HF ratio) were calculated on the basis of frequency domain analysis. HF power and LF/HF ratio were used for estimation of motion sickness severity as shown previously [3].

**Distortion product otoacoustic emissions (DPOAEs)**

DPOAEs (Supplemental Figure S6) were performed before and after unilateral exposure from the right ear to a pure tone of 85 dBZ at 100 Hz by a speaker of KSC-SW11 (KENWOOD, Japan) at a distance of 30 cm from the occipital area including the right inner ear. DPOAEs were detected by ER-10B (Etymotic Research Inc., Elk Grove Village, IL, USA), and data at 4 kHz were obtained. A DP-gram was measured as 2f1-f2 amplitudes, f1 and f2 were 65 dB and 55 dB sound pressure level (SPL), respectively, and f2/f1=1.2.

**References**

1. Boos CJ, Mellor A, Woods DR, O'Hara JP. The Effect of High-Altitude Acclimatisation on Ultra-Short Heart Rate Variability. Front Cardiovasc Med. 2022;9:787147. doi:10.3389/fcvm.2022.787147.

2. Chuang HW, Kao CW, Lee MD, Chang YC. Effectiveness of Story-Centred Care Intervention Program in older persons living in long-term care facilities: A randomized, longitudinal study. PLoS One. 2018;13:e0194178. doi:10.1371/journal.pone.0194178.

3. Lin CL, Jung TP, Chuang SW, Duann JR, Lin CT, Chiu TW. Self-adjustments may account for the contradictory correlations between HRV and motion-sickness severity. Int J Psychophysiol. 2013;87:70-80. doi:10.1016/j.ijpsycho.2012.11.003.

**Supplemental Figure Legends**

**Supplemental Figure S1. Movements of mice and human heads monitored by 6-axis motion sensors.** Accelerations in the X, Y, and Z directions and angular speed in roll, yaw and pitch in mice during shaking by two shakers (A) and in the heads of human subjects during shaking by a swing (B), a driving simulator (C) and a car (D) were recorded by using a 6-axis motion sensor.

**Supplemental Figure S2.** **Imbalance caused by a swing (SW) in human subjects without pure tone exposure.** A line graph for individual change in the ratio of the envelope area assessed by posturography before and after 1-min shaking of a swing is presented in the subjects without exposure to the pure tone. Significant difference (*** *P*<0.001) was analyzed by the paired *t*-test.

**Supplemental Figure S3. Effects of different pure tone exposures on motion sickness caused by a swing in humans.** (A) The experimental protocol for balance assessed by posturography in the absence or presence of 1-min exposure to a pure tone of 85 dBZ at 100 Hz just before 1-min shaking of a swing is presented. (B) Methods for unilateral exposure of a pure tone of 85 dBZ at 100 Hz to the right inner ear (R) and bilateral exposure of a pure tone of 85 dBZ at 250 Hz to both inner ears in the temporal bones are presented. (C, D) Changes in the ratio of the envelope area assessed by posturography before and after 1-min swing shaking in subjects with unilateral exposure to the pure tone at 100 Hz before the shaking (C, Uni-100 Hz) and subjects with bilateral exposure to the pure tone of 85 dBZ at 250 Hz before the shaking (D, Bi-250 Hz) were compared to those in the same subjects without sound exposure (C and C, No sound). Not significant differences (ns) were analyzed by the paired *t*-test.

**Supplemental Figure S4.** **Imbalance and autonomic dysregulation caused by a driving simulator in humans without pure tone exposure.**

(A, B) Line graphs for individual change in the ratio of the envelope area assessed by posturography (A) and individual change in the ratio of parasympathetic nerve activity (HF) and sympathetic nerve activity (LF/HF ratio) assessed by heart rate variability (HRV) before and after 3-min shaking of a driving simulator are presented for the subjects without exposure to the pure tone. Significant difference (*** *P*<0.001) was analyzed by the paired *t*-test.

**Supplemental Figure S5. Imbalance caused by a vehicle in human subjects without pure tone exposure.** A line graph for individual change in the ratio of the envelope area assessed by posturography before and after 3-min shaking of a vehicle is presented for the subjects without exposure to the pure tone. Significant difference (** *P*<0.01) was analyzed by the paired *t*-test.

**Supplemental Figure S6. Effects of exposure to a pure tone on hearing levels in humans.** (A) The method used for unilateral exposure of a pure tone of 85 dBZ at 100 Hz for 1 min toward the right inner ear (R) in the temporal bone is presented. (B) Hearing levels at 4,000 Hz in an exposed ear with (Uni-100 Hz) or without (No sound) exposure to the pure tone were evaluated by distortion product otoacoustic emissions (DPOAEs) in human subjects (n=9). Not significant differences (ns) were analyzed by the paired *t*-test. DPOAEs, distortion product otoacoustic emissions.

**Supplemental Table S1.** **List of subjects participated in different experiments.**

**Supplemental Table S2.** **Basic characteristics of participants.**
