# Supplementary material for: Just 1-min exposure to a pure tone at 100 Hz with daily exposable sound pressure levels may improve motion sickness
Source: Environ Health Prev Med. 2025 Mar 25;30:22. doi: 10.1265/ehpm.24-00247 (PMC11955832; doi:10.1265/ehpm.24-00247)
Supplement: Supplementary file 2 — Supplemental Figure S1. Movements of mice and human heads monitored by 6-axis motion sensors. Supplemental Figure S2. Imbalance caused by a swing (SW) in human subjects without pure tone exposure. Supplemental Figure S3. Effects of different pure tone exposures on motion sickness caused by a swing in humans. Supplemental Figure S4. Imbalance and autonomic dysregulation caused by a driving simulator in humans without pure tone exposure. Supplemental Figure S5. Imbalance caused by a vehicle in human subjects without pure tone exposure. Supplemental Figure S6. Effects of exposure to a pure tone on hearing levels in humans. Supplemental Table S1. List of subjects participated in different experiments. Supplemental Table S2. Basic characteristics of participants. [file ehpm-30-022-s001b.pptx]

## Slide 1
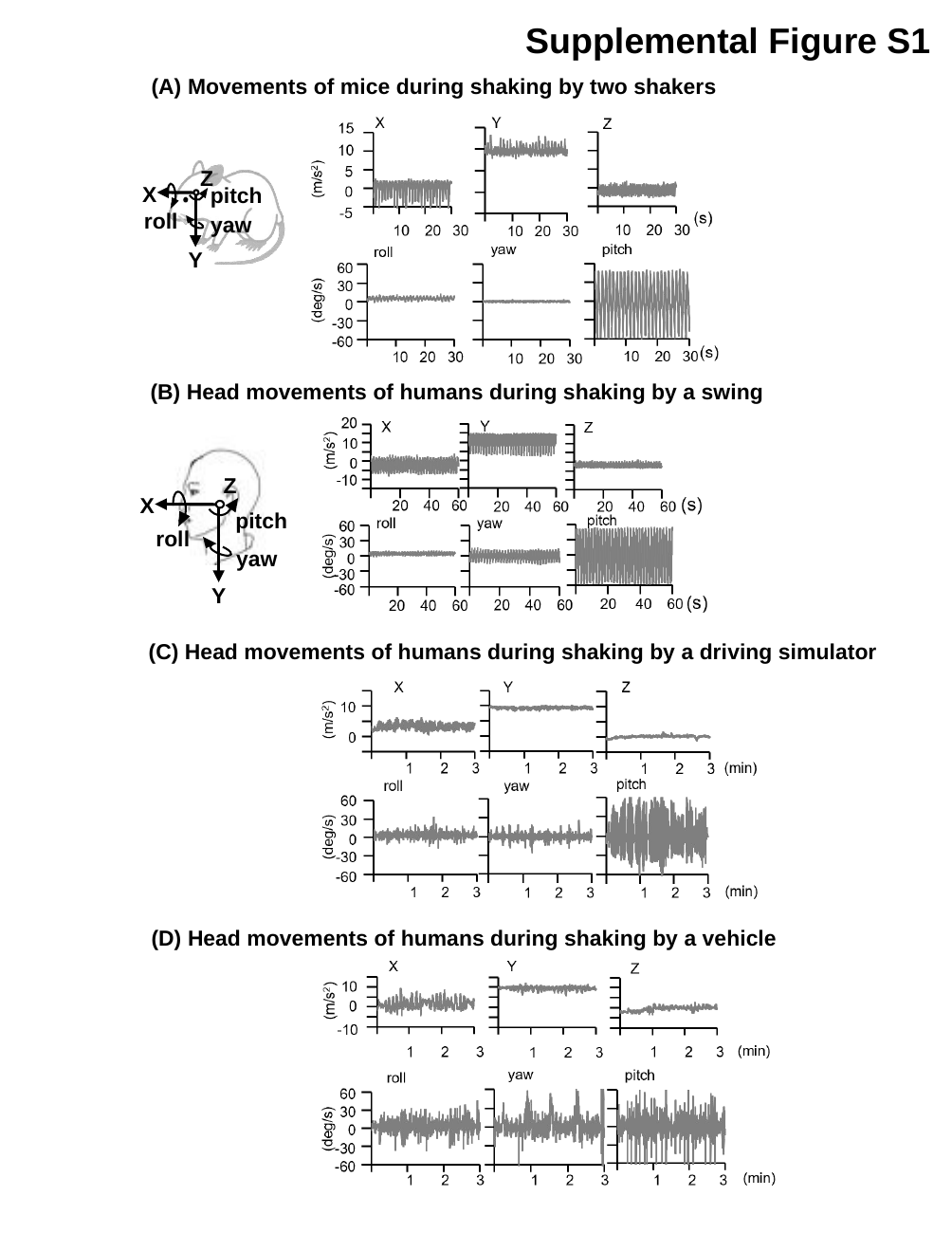

Supplemental Figure S1
(A) Movements of mice during shaking by two shakers
Z
X
pitch
roll
yaw
Y
(B) Head movements of humans during shaking by a swing
pitch
roll
yaw
Z
X
Y
(C) Head movements of humans during shaking by a driving simulator
(D) Head movements of humans during shaking by a vehicle

## Slide 2
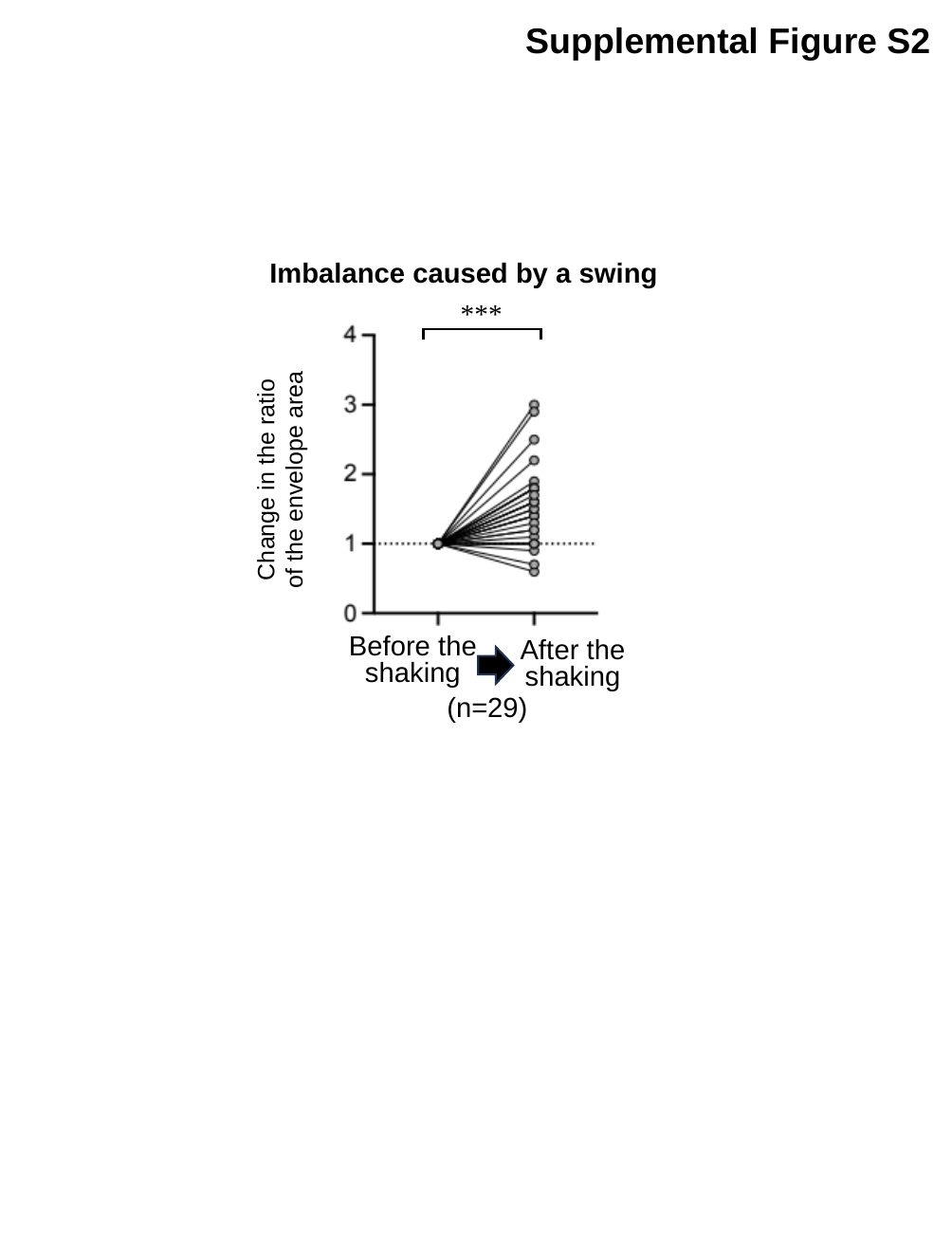

Supplemental Figure S2
Imbalance caused by a swing
***
Change in the ratio
of the envelope area
Before the shaking
After the shaking
(n=29)

## Slide 3
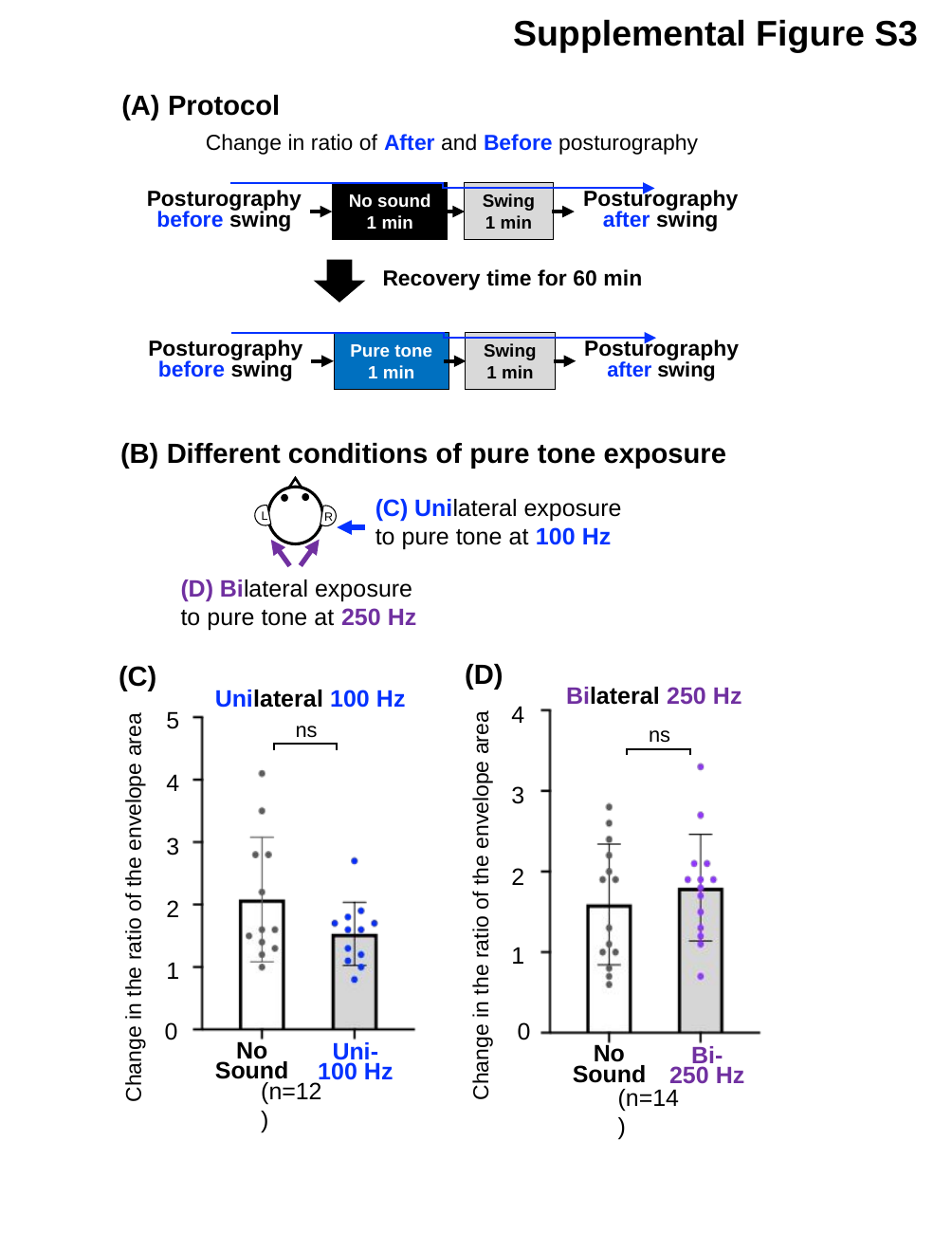

Supplemental Figure S3
(A) Protocol
Change in ratio of After and Before posturography
Posturography before swing
Posturography after swing
No sound
1 min
Swing
1 min
Recovery time for 60 min
Posturography before swing
Posturography after swing
Pure tone
1 min
Swing
1 min
(B) Different conditions of pure tone exposure
L
R
(C) Unilateral exposure
to pure tone at 100 Hz
(D) Bilateral exposure
to pure tone at 250 Hz
(D)
(C)
Bilateral 250 Hz
Unilateral 100 Hz
4
3
2
1
0
5
4
3
2
1
0
ns
ns
Change in the ratio of the envelope area
Change in the ratio of the envelope area
No
Sound
Uni-100 Hz
(n=12)
No
Sound
Bi-
250 Hz
(n=14)

## Slide 4
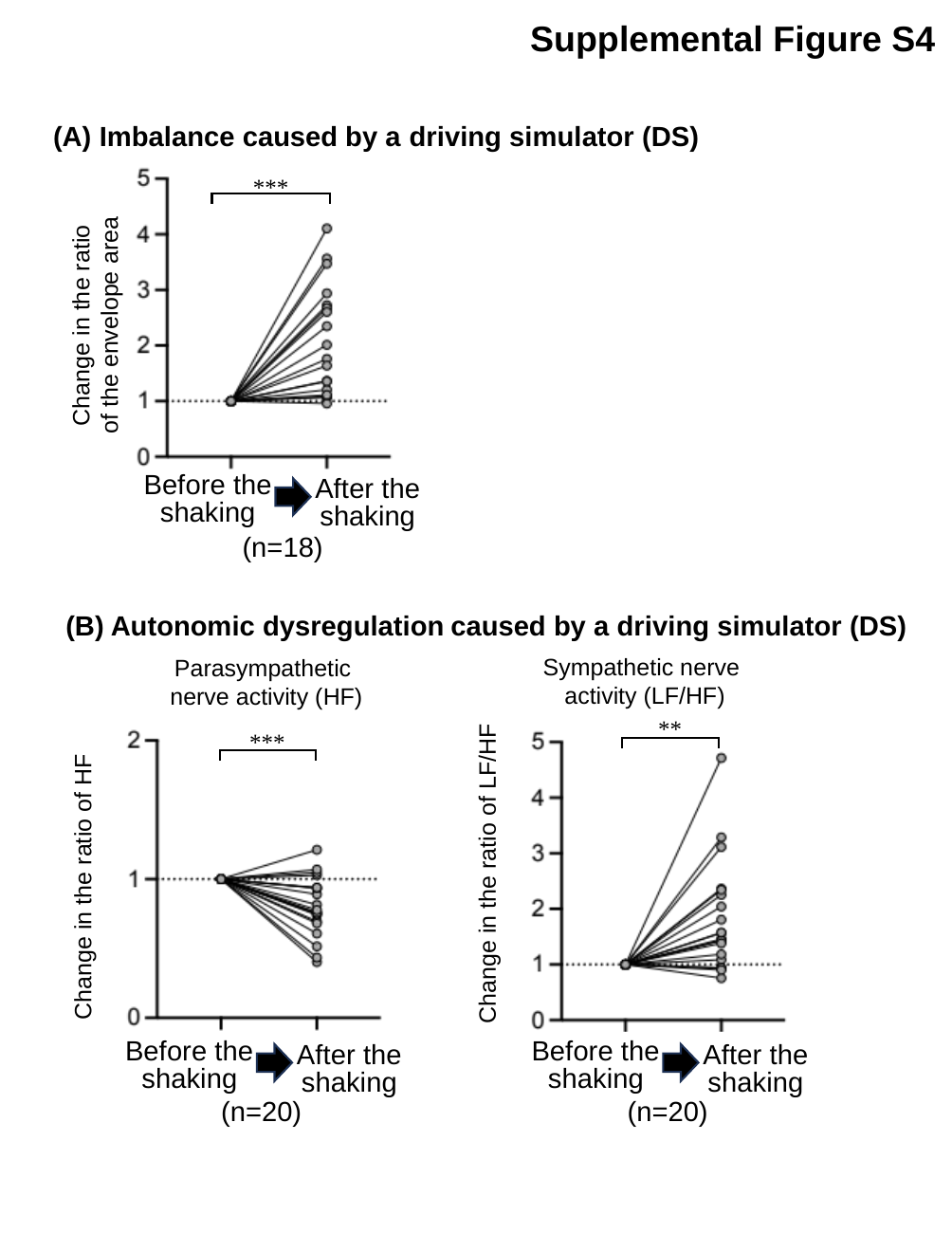

Supplemental Figure S4
***
Change in the ratio
of the envelope area
(A) Imbalance caused by a driving simulator (DS)
Before the shaking
After the shaking
(n=18)
(B) Autonomic dysregulation caused by a driving simulator (DS)
Sympathetic nerve
activity (LF/HF)
Parasympathetic
nerve activity (HF)
**
***
Change in the ratio of LF/HF
Change in the ratio of HF
Before the shaking
After the shaking
(n=20)
Before the shaking
After the shaking
(n=20)

## Slide 5
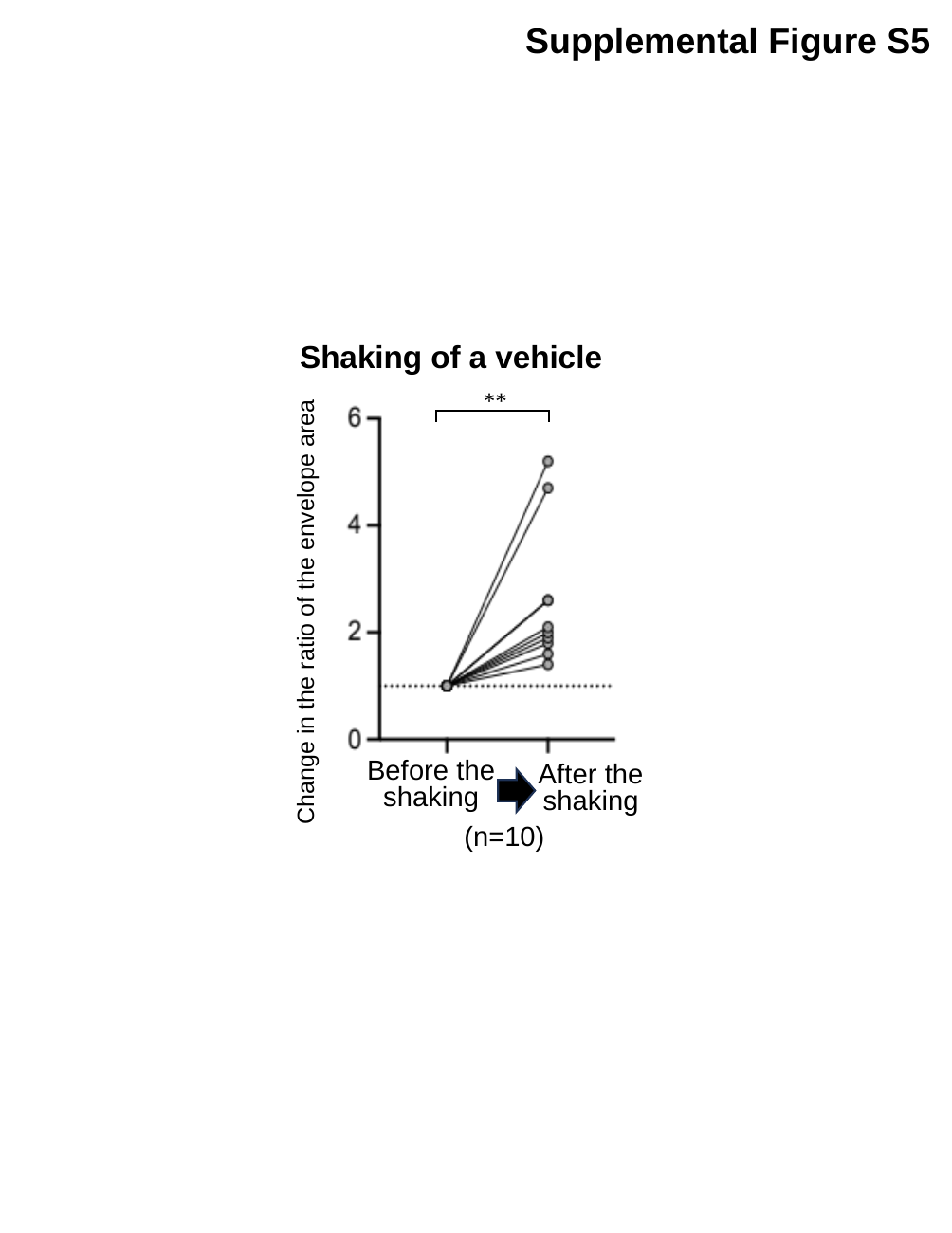

Supplemental Figure S5
Shaking of a vehicle
**
Change in the ratio of the envelope area
Before the shaking
After the shaking
(n=10)

## Slide 6
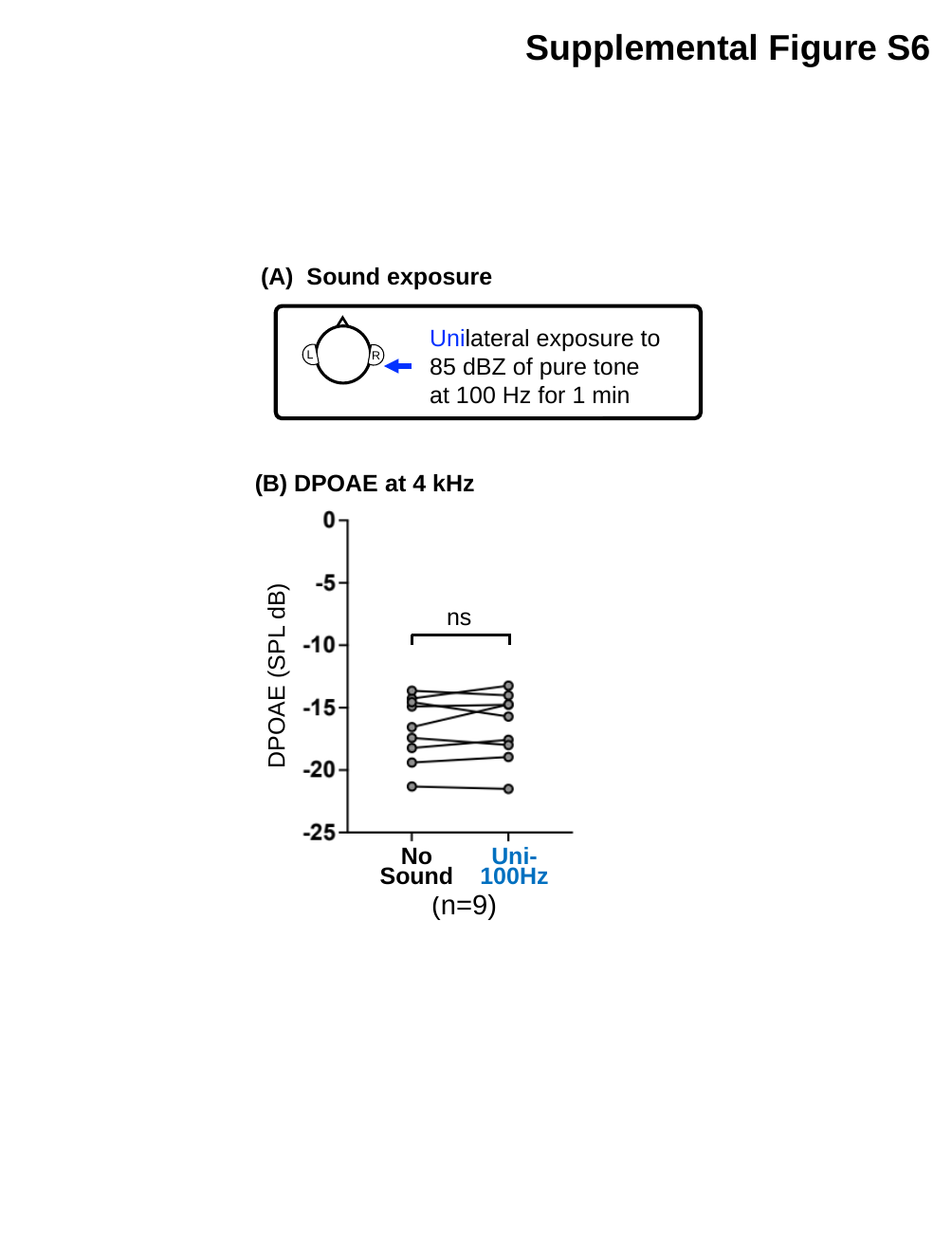

Supplemental Figure S6
(A) Sound exposure
Unilateral exposure to 85 dBZ of pure tone at 100 Hz for 1 min
L
R
(B) DPOAE at 4 kHz
ns
DPOAE (SPL dB)
No
Sound
Uni-
100Hz
(n=9)

## Slide 7
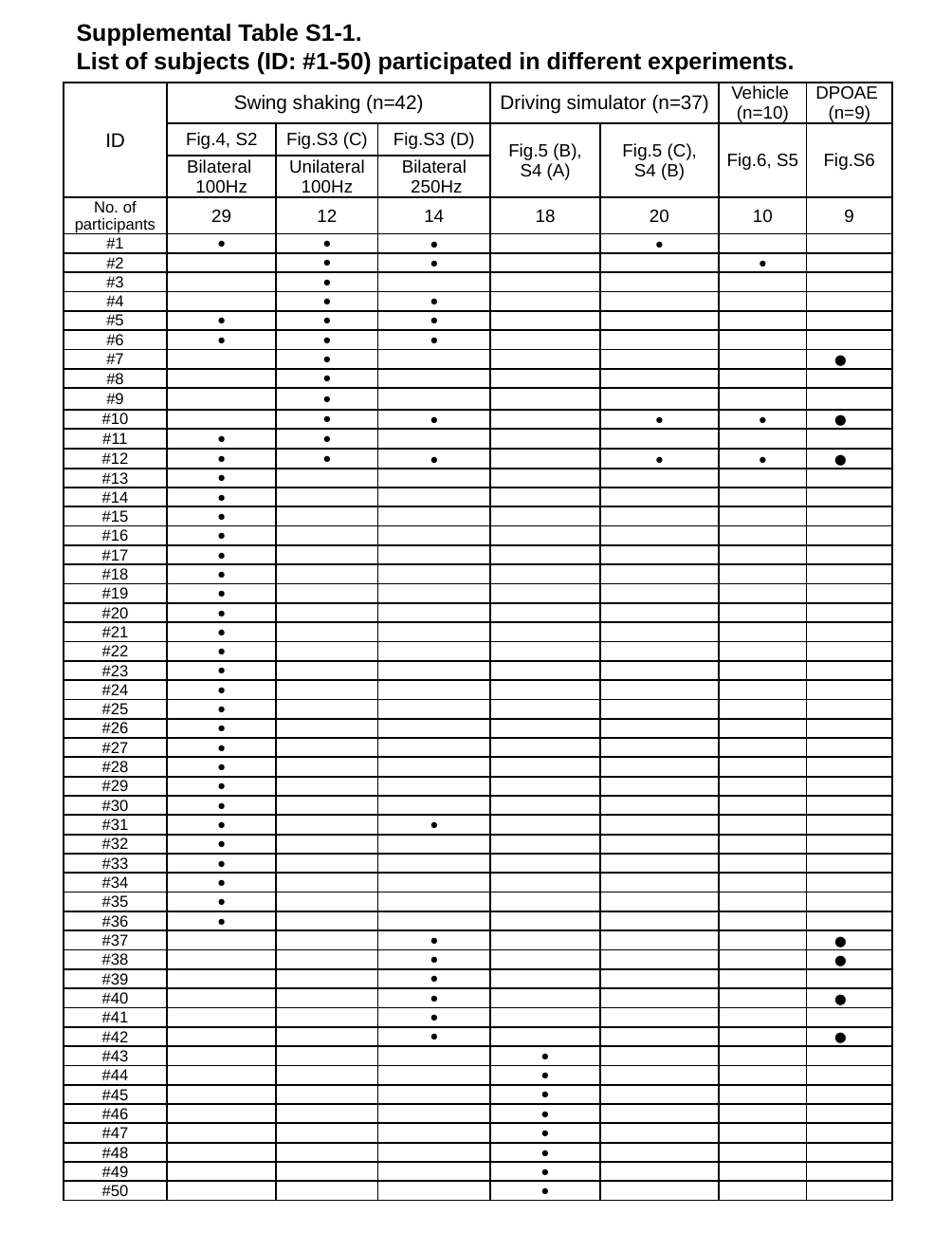

Supplemental Table S1-1.
List of subjects (ID: #1-50) participated in different experiments.
| ID | Swing shaking (n=42) | | | Driving simulator (n=37) | | Vehicle (n=10) | DPOAE (n=9) |
| --- | --- | --- | --- | --- | --- | --- | --- |
| | Fig.4, S2 | Fig.S3 (C) | Fig.S3 (D) | Fig.5 (B), S4 (A) | Fig.5 (C), S4 (B) | Fig.6, S5 | Fig.S6 |
| | Bilateral 100Hz | Unilateral 100Hz | Bilateral 250Hz | | | | |
| No. of participants | 29 | 12 | 14 | 18 | 20 | 10 | 9 |
| #1 | ● | ● | ● | | ● | | |
| #2 | | ● | ● | | | ● | |
| #3 | | ● | | | | | |
| #4 | | ● | ● | | | | |
| #5 | ● | ● | ● | | | | |
| #6 | ● | ● | ● | | | | |
| #7 | | ● | | | | | ● |
| #8 | | ● | | | | | |
| #9 | | ● | | | | | |
| #10 | | ● | ● | | ● | ● | ● |
| #11 | ● | ● | | | | | |
| #12 | ● | ● | ● | | ● | ● | ● |
| #13 | ● | | | | | | |
| #14 | ● | | | | | | |
| #15 | ● | | | | | | |
| #16 | ● | | | | | | |
| #17 | ● | | | | | | |
| #18 | ● | | | | | | |
| #19 | ● | | | | | | |
| #20 | ● | | | | | | |
| #21 | ● | | | | | | |
| #22 | ● | | | | | | |
| #23 | ● | | | | | | |
| #24 | ● | | | | | | |
| #25 | ● | | | | | | |
| #26 | ● | | | | | | |
| #27 | ● | | | | | | |
| #28 | ● | | | | | | |
| #29 | ● | | | | | | |
| #30 | ● | | | | | | |
| #31 | ● | | ● | | | | |
| #32 | ● | | | | | | |
| #33 | ● | | | | | | |
| #34 | ● | | | | | | |
| #35 | ● | | | | | | |
| #36 | ● | | | | | | |
| #37 | | | ● | | | | ● |
| #38 | | | ● | | | | ● |
| #39 | | | ● | | | | |
| #40 | | | ● | | | | ● |
| #41 | | | ● | | | | |
| #42 | | | ● | | | | ● |
| #43 | | | | ● | | | |
| #44 | | | | ● | | | |
| #45 | | | | ● | | | |
| #46 | | | | ● | | | |
| #47 | | | | ● | | | |
| #48 | | | | ● | | | |
| #49 | | | | ● | | | |
| #50 | | | | ● | | | |

## Slide 8
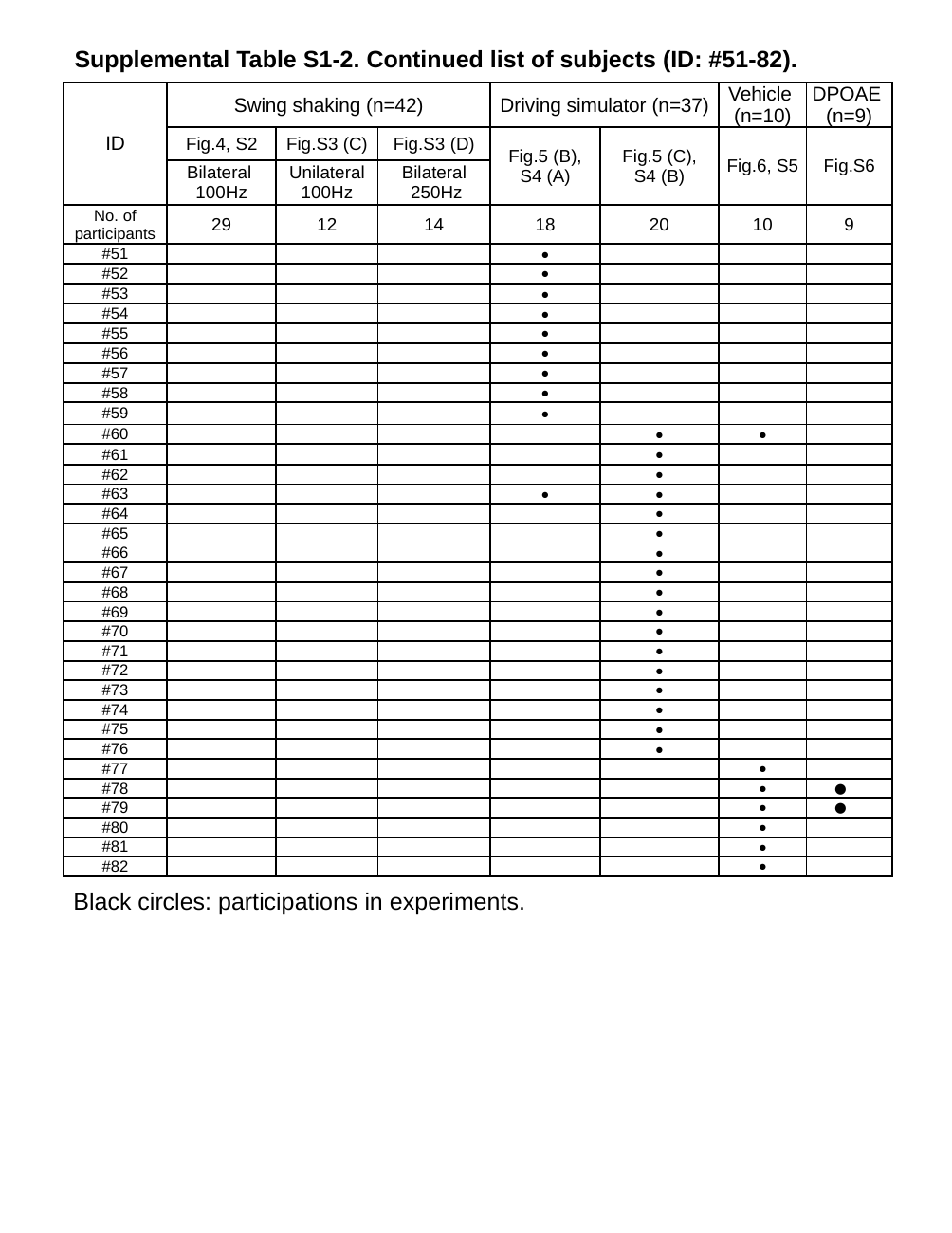

Supplemental Table S1-2. Continued list of subjects (ID: #51-82).
| ID | Swing shaking (n=42) | | | Driving simulator (n=37) | | Vehicle (n=10) | DPOAE (n=9) |
| --- | --- | --- | --- | --- | --- | --- | --- |
| | Fig.4, S2 | Fig.S3 (C) | Fig.S3 (D) | Fig.5 (B), S4 (A) | Fig.5 (C), S4 (B) | Fig.6, S5 | Fig.S6 |
| | Bilateral 100Hz | Unilateral 100Hz | Bilateral 250Hz | | | | |
| No. of participants | 29 | 12 | 14 | 18 | 20 | 10 | 9 |
| #51 | | | | ● | | | |
| #52 | | | | ● | | | |
| #53 | | | | ● | | | |
| #54 | | | | ● | | | |
| #55 | | | | ● | | | |
| #56 | | | | ● | | | |
| #57 | | | | ● | | | |
| #58 | | | | ● | | | |
| #59 | | | | ● | | | |
| #60 | | | | | ● | ● | |
| #61 | | | | | ● | | |
| #62 | | | | | ● | | |
| #63 | | | | ● | ● | | |
| #64 | | | | | ● | | |
| #65 | | | | | ● | | |
| #66 | | | | | ● | | |
| #67 | | | | | ● | | |
| #68 | | | | | ● | | |
| #69 | | | | | ● | | |
| #70 | | | | | ● | | |
| #71 | | | | | ● | | |
| #72 | | | | | ● | | |
| #73 | | | | | ● | | |
| #74 | | | | | ● | | |
| #75 | | | | | ● | | |
| #76 | | | | | ● | | |
| #77 | | | | | | ● | |
| #78 | | | | | | ● | ● |
| #79 | | | | | | ● | ● |
| #80 | | | | | | ● | |
| #81 | | | | | | ● | |
| #82 | | | | | | ● | |
Black circles: participations in experiments.

## Slide 9
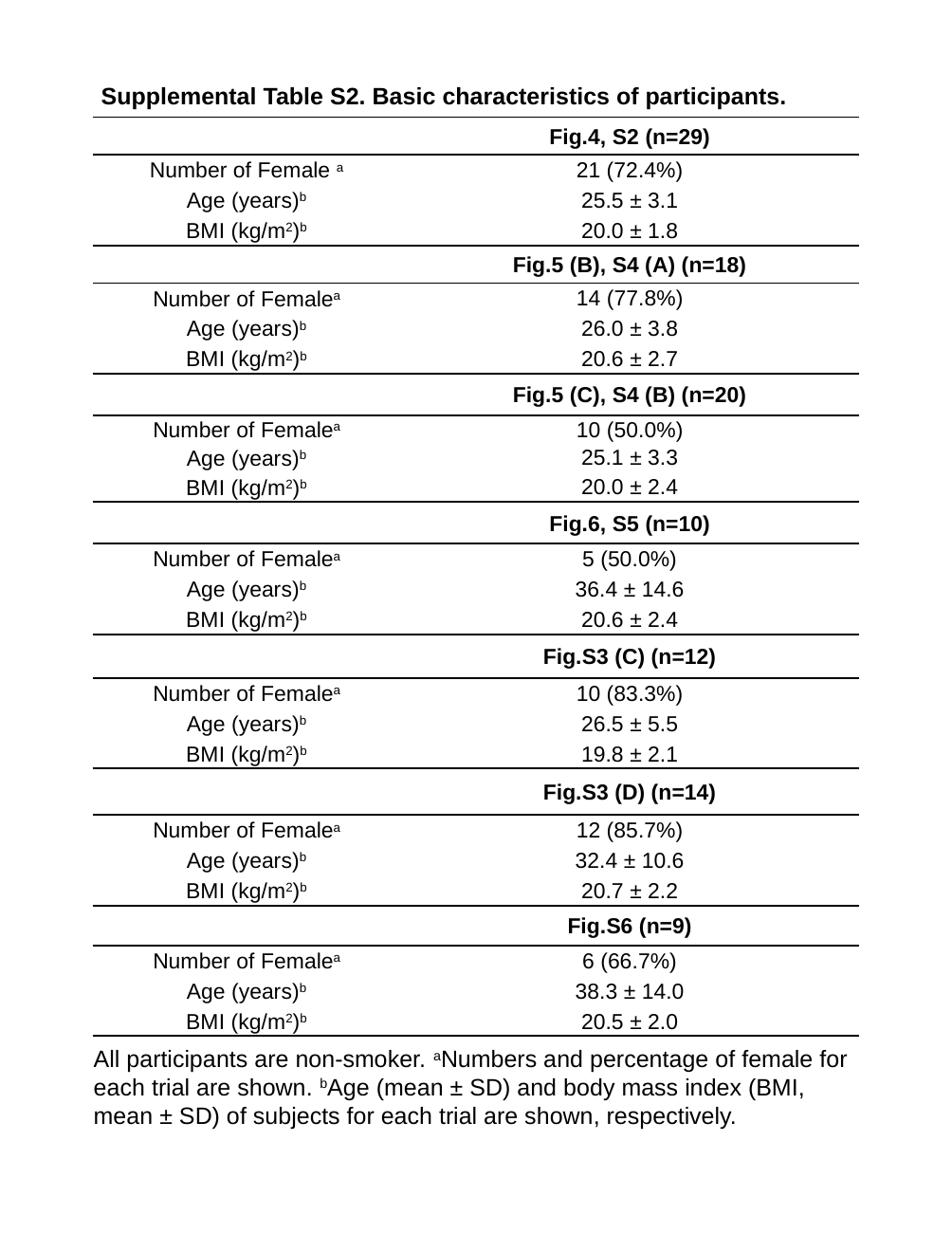

Supplemental Table S2. Basic characteristics of participants.
| | Fig.4, S2 (n=29) |
| --- | --- |
| Number of Female a | 21 (72.4%) |
| Age (years)b | 25.5 ± 3.1 |
| BMI (kg/m2)b | 20.0 ± 1.8 |
| | Fig.5 (B), S4 (A) (n=18) |
| Number of Femalea | 14 (77.8%) |
| Age (years)b | 26.0 ± 3.8 |
| BMI (kg/m2)b | 20.6 ± 2.7 |
| | Fig.5 (C), S4 (B) (n=20) |
| Number of Femalea | 10 (50.0%) |
| Age (years)b | 25.1 ± 3.3 |
| BMI (kg/m2)b | 20.0 ± 2.4 |
| | Fig.6, S5 (n=10) |
| Number of Femalea | 5 (50.0%) |
| Age (years)b | 36.4 ± 14.6 |
| BMI (kg/m2)b | 20.6 ± 2.4 |
| | Fig.S3 (C) (n=12) |
| Number of Femalea | 10 (83.3%) |
| Age (years)b | 26.5 ± 5.5 |
| BMI (kg/m2)b | 19.8 ± 2.1 |
| | Fig.S3 (D) (n=14) |
| Number of Femalea | 12 (85.7%) |
| Age (years)b | 32.4 ± 10.6 |
| BMI (kg/m2)b | 20.7 ± 2.2 |
| | Fig.S6 (n=9) |
| Number of Femalea | 6 (66.7%) |
| Age (years)b | 38.3 ± 14.0 |
| BMI (kg/m2)b | 20.5 ± 2.0 |
All participants are non-smoker. aNumbers and percentage of female for each trial are shown. bAge (mean ± SD) and body mass index (BMI, mean ± SD) of subjects for each trial are shown, respectively.
